# Multidimensional analyses of the noise impacts of COVID-19 lockdowna)

**DOI:** 10.1121/10.0009324

**Published:** 2022-02-09

**Authors:** Pierre Aumond, Arnaud Can, Mathieu Lagrange, Felix Gontier, Catherine Lavandier

**Affiliations:** 1 Joint Research Unit in Environmental Acoustics/Unité Mixte de Recherche en Acoustique Environnementale, Univ Gustave Eiffel, Institut français des sciences et technologies des transports, de l'aménagement et des réseaux (IFSTTAR), Centre d'études et d'expertise sur les risques, l'environnement, la mobilité et l'aménagemen Bouguenais, F-44344, France; 2 Laboratoire des Sciences du Numérique de Nantes (LS2N), Unité Mixte de Recherche Centre national de la recherche scientifique 6004, Ecole Centrale de Nantes Nantes, F-44321, France; 3 Equipes Traitement de l'Information et Systéme UMR 8051, CY Cergy Paris Univ, École Nationale Supérieure de L'électronique et de ses Applications, CNRS, Cergy-Pontoise, F-95000, France

## Abstract

As part of the Agence Nationale de Recherche Caractérisation des ENvironnements SonorEs urbains (Characterization of urban sound environments) project, a questionnaire was sent in January 2019 to households in a 1 km^2^ study area in the city of Lorient, France, to which about 318 responded. The main objective of this questionnaire was to collect information about the inhabitants' perception of the sound environments in their neighborhoods, streets, and dwellings. In the same study area, starting mid-2019, about 70 sensors were continuously positioned, and 15 of them were selected for testing sound source recognition models. The French lockdown due to the COVID-19 crisis occurred during the project, and the opportunity was taken to send a second questionnaire during April 2020. About 31 of the first 318 first survey respondents answered this second questionnaire. This unique longitudinal dataset, both physical and perceptual, allows the undertaking of an analysis from different perspectives of such a period. The analysis reveals the importance of integrating source recognition tools, soundscape observation protocol, in addition to physical level analysis, to accurately describe the changes in the sound environment.

## I. INTRODUCTION

The emergence and the spread of the COVID-19 pandemic from late 2019 to 2020 impacted all continents. It forced governments to undertake unprecedented social distancing measures to slow down the virus propagation, from which the most emblematic was the lockdown imposed in a large number of countries in the spring of 2020. Severe restrictions on ground transportation and flights, as well as population lockdown measures, had an immediate and dramatic impact on urban activity and, thus, on sound environments. City dwellers in many cities around the world have collectively experienced a sudden reduction of noise levels, including a modification in the distribution of sound sources dominating urban sound mixtures. Newspapers quickly reported on these sudden changes in the urban sound environments and their impact on perceptions (Bui and Badger, 2020). From a research point of view, this unprecedented event questions the ways in which the physical modifications in urban sound environments during this period can be witnessed and objectified and their perception by the populations. This question is crucial to understand the impact of such a crisis, which is likely to modify perceptions and expectations regarding noise in the long term. It is also important to propose protocols, which are able to capture and understand the impact of slower and less obvious modifications in sound environments.

Despite initiatives to homogenize observations, such as the one proposed by Asensio *et al.* (2020a), the observation protocols in the recent literature on the topic of the change of the sound environment resulting from the COVID-19 lockdown are very disparate. We have chosen to refer to about 17 studies, which we consider to be the most representative.

First, cities equipped with noise measurement networks, capturing continuous noise levels, have been quick to consistently point out the drop in the noise levels. An equivalent sound level reduction of about 4–7 dB(A) has been reported, on average, in Rio (Gevú *et al.*, 2021), Montreal (Steele and Guastavino, 2021), Lyon (Munoz *et al.*, 2020), Madrid (Asensio *et al.*, 2020b), Milan (Zambon *et al.*, 2021), Girona (Alsina-Pagès *et al.*, 2021), or Paris (Bruitparif, 2020) based on 21 to more than 100 fixed sensors. Numerous short-term measurements were performed in London in Aletta *et al.* (2020) through a series a 30-s binaural recordings pre-lockdown and during the lockdown (481 samples) at 11 locations, which highlighted a similar tendency. This sound level decrease is, however, not homogeneous in both space and time. The London study showed that active areas were affected the most, followed by traffic-dominated areas and quiet areas. Similar trends were found in Madrid, although less pronounced (Asensio *et al.*, 2020b), or the Brazilian study (Gevú *et al.*, 2021), which complements its measurements with the modeling approach. These slight discrepancies between the two studies may be the result of a different classification between the site categories. The observed noise decrease has even reached 20 dB(A) near the work site areas and 30 dB(A) near the airports in Paris (Bruitparif, 2020), revealing the difference in the “lockdown sound experience” between populations. In Montreal, special emphasis is being placed on dramatically reducing noise at festivals and events in public spaces during the summer of 2020 (Steele and Guastavino, 2021). In the temporal structures of sound, environments were also impacted. Basu *et al.* (2021) showed that minimum hourly sound levels L_min,1h_ dramatically decreased, which were attributed to the reductions in road and air traffic movements. In Madrid, Asensio *et al.* (2020b) showed a significant variation in the daily noise patterns with the activity starting earlier in the morning and decreasing significantly in the afternoon.

Although very useful for understanding which populations were most impacted by the reduction of noise levels based on their residential location and on a national scale based on government decisions, these studies fail to qualify the modification of noise environments in terms of the sound sources. The shift in daily noise patterns observed in Asensio *et al.* (2020b) could, for instance, be hypothesized as the result of the emergence in natural sound sources. Derryberry *et al.* (2020) showed that white-crowned sparrows shifted their song frequency in response to the disappearance of traffic sounds, benefiting this new emptied acoustic space to enhance communication. These results underline the importance of being able to recognize the sources composing the sound mixtures within the sensor networks for a better understanding of the balances between the anthropogenic and biophonic sound sources. The Dynamap project in Italy also studied the differences in the sound environment caused by the lockdown due to COVID-19 in Italy (period 2019 vs 2020; Pagès *et al.*, 2020). Their ANED (anomalous noise events detection) algorithm identifies the non-traffic-related sounds using the binary identifier classifications. It showed a distinct change in anomalous noise events (ANEs) during the night in Rome and, to a lesser extent, Milan.

The studies based on perceptual approaches and questionnaires have also underlined the impact of the lockdown on the perceived sound environments. In Argentina, a study of 1000 people showed that most participants preferred the new acoustic environment and, especially in large cities, where mechanical sounds dominate the sound environment (Maggi *et al.*, 2021). The analyses of noise complaints in London showed an increase of 48%, which is mainly because of construction and neighborhood noise (Tong *et al.*, 2021). In Munoz *et al.* (2020), Bruitparif (2020), and Bartalucci *et al.* (2021), the questionnaires were distributed to residents in the French and Italian countries. The analyses of those questionnaires underlined the perceived modification in the sound environments, namely, a decrease in the transportation and mechanical sound sources and an increase in the natural sound sources. The questions relative to the period before and during the lockdown period were answered simultaneously. This could have introduced memory and cognitive biases. Finally, Lenzi *et al.* (2021) provided a comprehensive analysis of the sound environment at one location in the city of Gexto, based on audio recordings and annotations of the perceived sounds, diary notes, and evaluation of the soundscape quality. The study revealed that bioacoustic indices, such as eventfulness, acoustic complexity, and acoustic richness, increased significantly over the lockdown period, whereas the amount of technological sounds decreased.

Although studies on noise levels, on one hand, and studies on perception, on the other hand, have their respective merits, we believe that performing both at the same time and location can shed a new light on the topic under study. For that purpose, in this paper, an innovative protocol with a great level of detail is proposed to relate the physical and perceptual modifications of the sound environments during this period. It associates a measurement network coupled with an automatic sound source recognition module and questionnaires distributed before and during the lockdown. The objective here is not to define a universal characterization of the impact of the lockdown but to demonstrate the relevance of such a protocol to characterize such an event. The protocol of this study follows closely the recommendations described in the “triangulation” section of the ISO/TS (2019) soundscape standard and aims to test/demonstrate its value.

This paper is organized as follows. Section II presents the questionnaires and measurement network, which includes a sound recognition module. Section III presents the perceptual analysis as well as the analyses of the sound levels and the perceived times of presence for the different sound sources. This section finishes with a cross-analysis of those indicators. A discussion of these results is then given in Sec. IV.

## II. MATERIAL AND METHODS

### A. Questionnaire

During the second week of January 2019, a questionnaire was sent to approximately 2000 households in a 1 km^2^ wide study area in the city of Lorient, France. Until March 15, 2019, the residents were allowed to return a paper version of the questionnaire or complete it through a website platform. The questionnaire was designed to take about 20–25 min to complete and is composed of five sections detailed below. A second questionnaire was sent to pthe articipants of the first questionnaire during the lockdown period in 2020 from early April until mid May. It was identical in every aspect concerning the first two sections. Of these participants, 318 people completed the first questionnaire and about 50 people also completed the second questionnaire (31 complete questionnaires).

In the first section of the questionnaire, the respondents had to assess the quality of the sound environment in their neighborhood and then in their street (when walking or cycling home). The evaluation relied on five bipolar semantic scales (seven levels) inspired by the Swedish protocol (Axelsson *et al.*, 2012). Table I presents the French semantic elements as well as a proposal for the translation into English.

**TABLE I.** The elements of the bipolar scales (1–7). The last column corresponds to their codification.

*Désagréable*	Unpleasant	*Agréable*	Pleasant	Pl
*Inerte*, *amorphe*	Inert	*Animé, mouvementé*	Eventful	Ev
*Bruyant*	Noisy	*Silencieux*	Silent	Si
*Ennuyeux*, *inintéressant*	Boring	*Stimulant, intéressant*	Exciting	Ex
*Agité*, *chaotique*	Chaotic	*Calme, tranquille*	Calm	Ca
*En inadéquation avec vos attentes*	In inadequacy with your expectations	*En adéquation avec vos attentes*	In adequacy with your expectations	Ad

Then the respondents had to fill in a table dedicated to the perceived time of presence and the perceived sound level of 13 sound sources, which they could have heard when they came in or out of their homes, on foot or by bike, on their streets, and during the year (long-term assessment). The perceived time of the presence ranges from rarely or never present (“1”) to always present (“7”) in the sound environment. For the latter, they had the possibility to mention the season when the source was specifically heard. The nomenclature had been previously established using information from sound sources *in situ*, bibliographic work, and their own previous studies (Aumond *et al.*, 2017; Ricciardi *et al.*, 2015). Table II presents the sources that were assessed. A free comment window closed this first section, allowing the respondent to give more details about their perceptions.

**TABLE II.** The list of sources that were assessed in the questionnaire.

Road traffic (Tra)	Sirens, alarms (Sir)	Children's voices (schools, playgrounds; ChV)	Gulls^a^ (Gul)
Two-wheeler (2Wh) motor vehicles	Urban maintenance (cleaning, garbage, etc.; UMa)	Music from bars, restaurants, shops, etc. (Mus)	Sources from neighboring dwellings (voices, steps, animals, crafts, music, etc.)
Rail traffic (rail)	Expressive voices, festive voices, laughter, shouts (ExV)	Wind in the vegetation (Wnd)	Other
Air traffic (air)	Calm voices, conversations, etc. (CaV)	Small birds (Brd)	Other

^a^ Lorient is a harbor city with several complaints in the local press about the noise of gulls.

The second section of the questionnaire focused on the long-term annoyance. The respondents were asked questions on the annoyance, following the guidelines from the noise team of ICBEN Fields *et al.*, 2001). This section of the questionnaire can be summarized by the following sentence:

“Thinking about the last 12 months, when you are here

• at home with your windows closed,
• at home with your windows open, on your balcony, in your garden, or
• in the street when you arrive at home by bike or on foot,

how much does

• global noise and
• the noise sources from Table II

bother, disturb, or annoy you: extremely, very, moderately, slightly, or not at all?

In the third section of the questionnaire, four free paragraph boxes allowed the respondents to share free expressions regarding the remarkable environments (pleasant, unpleasant, conducive to walking, and conducive to rest) of their neighborhood.

In the fourth section of the questionnaire, personal information was collected: noise sensitivity of the inhabitants based on the six-item Weinstein's noise sensitivity scale (WNSS; Kishikawa *et al.*, 2006), gender, age, socio-professional category, and membership (or not) to an association fighting against noise.

In the fifth and last section of the questionnaire, the residents were invited to provide information on where they live: the exact location, such that the questionnaires can be linked with the acoustic measurements or simulations made in the area, and complete a set of questions on the housing type (Table III).

**TABLE III.** Questions about the housing of the participants.

Tenant/owner	Courtyard or garden area? (yes/no)	Has a quiet room? (yes /no)
House/apartment	Living space overlooking the street? (yes/no)	Double glazing? (yes /no)
Time of occupancy? (<1 yr, 1–3 yr, >3 yr)	Living space with a view on natural elements? (no, a little, a lot)	Insulation of the facade <10 years ago? (yes/no)

Under the fifth section, the respondents also had to give their level of satisfaction (five levels) on four dimensions:

• acoustic insulation of their housing and
• to what extent they are globally satisfied with their (home/street/neighborhood) as a place to live.

More information can be found in the conference papers by Aumond *et al.* (2019) and Aumond and Lavandier (2019).

### B. Measurement network

Specific low-cost noise monitoring sensors have been developed by the CENSE project to be integrated in a large measurement network (Ardouin *et al.*, 2021; Picaut *et al.*, 2021). The complete network planned is 123 noise sensors with 70 sensors currently connected to the cloud through a hybrid communication network based on the wireless and public street lamp network equipped with power-line communication systems. The sensors have been specifically designed and developed to consider the urban sound environment constraints. They transmit acoustic indicators continuously, thanks to the wireless communications based on the 802.15.4 modulation with a IPv6 Low power Wireless Personal Area Networks (6LoWPAN) Medium Access Control (MAC) layer as described by IEEE (Montenegro, 2007). The microphones used are micro-electromechanical system (MEMS) microphones, and the recording and transmitting systems are based on the STM32L4 microcontrollers or small single-board computers Raspberry-Pi. Real-time audio processing is included in both types of sensors to perform the calculations of the L_Aeq,1s_ and L_Zeq,1s_ acoustic indicators, as well as the acoustic spectrum every 125 ms, using the third-octave bands from 20 Hz to 12.5 kHz. The recording sampling rate is 32 kHz.

**FIG. 1.**
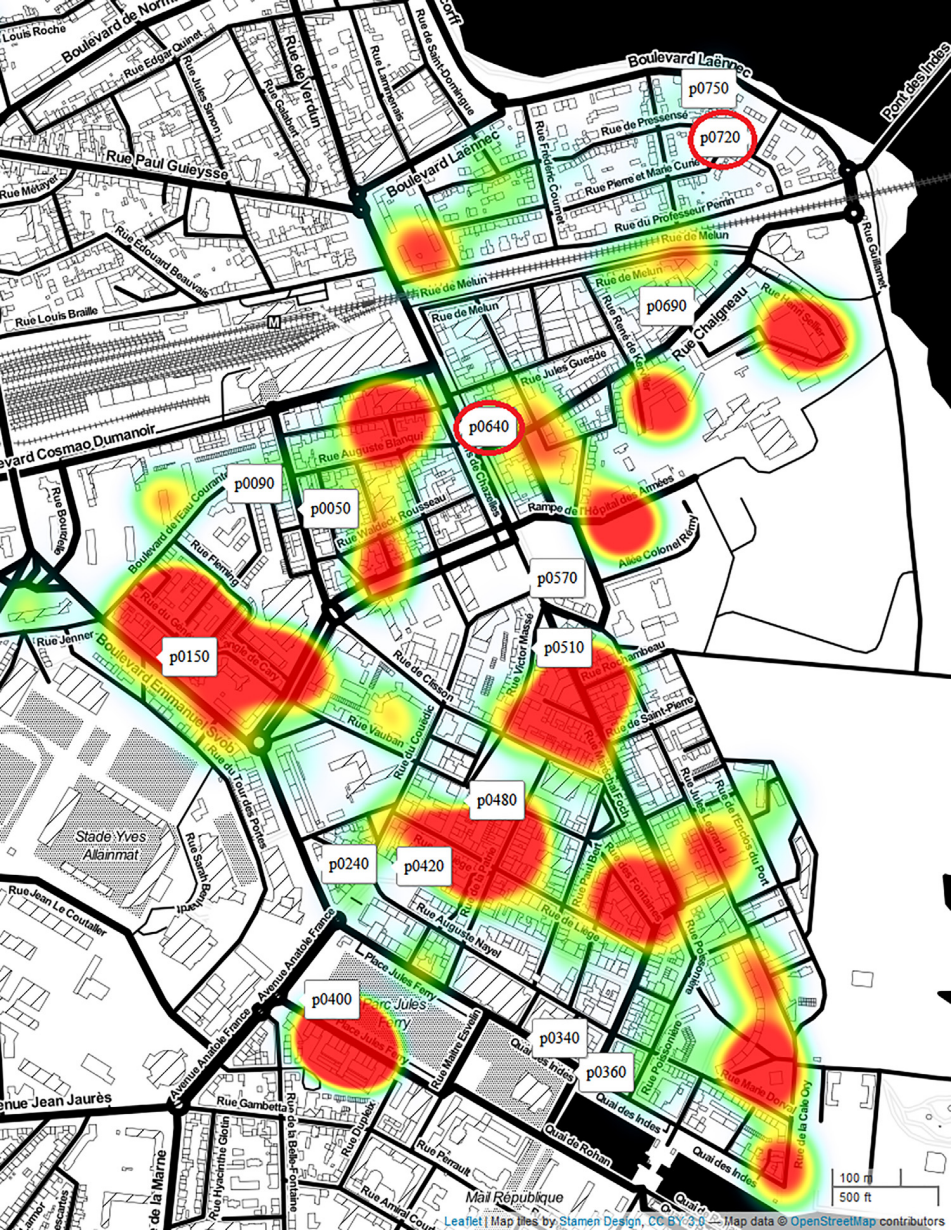
(Color online) The CENSE study area. The labels correspond to the selected sensors (*n* = 15). The labels circled in red correspond to the stations that we focus on more during the analysis. The heat map displays the density of the responses to the questionnaire in the first call (*n* = 318).

In April 2020, about 70 sensors were installed in the study area. Among these sensors, we strategically selected 15 that reliably transmitted data over the study periods and are spatially distributed to match the areas of living for the respondents of the questionnaires. To facilitate the processing of the data, only the first 10 min of each hour are analysed under the assumption suggested in Brocolini *et al.* (2013), and they are representative of the entire homogeneous corresponding period. Figure 1 shows the comparison between the locations of the measurement points (labeled pins) and the spatial distribution of the responses to the questionnaires (heatmap).

### C. Sound recognition

In addition to the analyses of the subjective assessments and acoustic indicators, we investigated the variations in the content of the sound environments through automatic sound source recognition. Specifically, a deep neural network has been designed to identify the cars, trucks, motorcycles, voices, small birds, seagulls, and background noise activities from the CENSE sensor measurements.

Source identification is conducted on short segments of eight fast third-octave frames (8 × 125 ms = 1 s). To do so, a deep convolutional architecture first extracts the time-frequency patterns, which are relevant to the identification of the sound sources, from each 1 s third-octave segment. It is composed of 6 layers with 3 × 3 filters and 64, 64, 128, 128, 256, and 256 output channels. The convolutional layers are followed by batch normalization (Ioffe and Szegedy, 2015) and rectified linear unit activations. The maximum pooling layers down sample the hidden representation in the time and frequency by a factor of 2 after each set of two convolutional layers. Then, a single-layer gated recurrent unit (Cho *et al.*, 2014) with 128 neurons draws the predictions on each 1 s segment from the current output of the convolutional architecture as well as its recurrent internal state, which aggregates the past information. The step duration between the subsequent 1 s segments processed by the network is 125 ms. At the inference, presence or absence labels predicted for each sound source are averaged over time to obtain the time of presence in the third-octave measurements of arbitrary duration.^1^

**FIG. 2.**
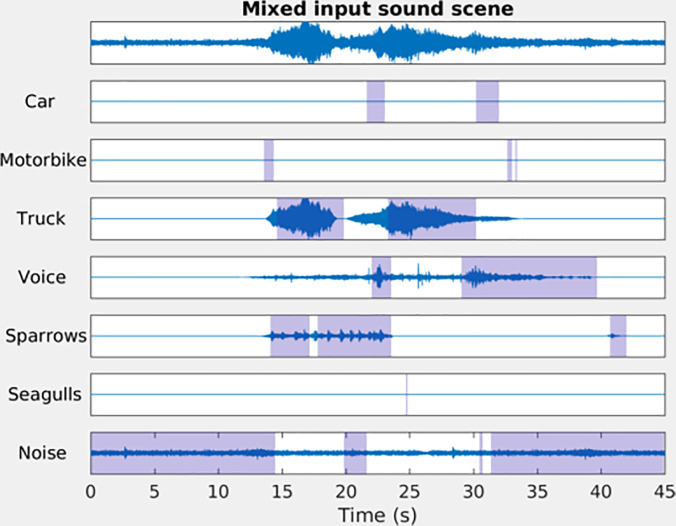
(Color online) An example of the predicted source activity for a simulated sound scene. From the mixed scene (top), the model infers the presence of seven sound sources along the time (shaded areas). The separated waveform contributions for each source are shown for illustrative purposes and hidden to the predictor.

The model is trained on a fully synthetic set of 400 sound scenes of 45 s each as described in Gontier *et al.* (2021). The sound scenes are simulated with the simScene MATLAB library^2^ by combining the background noise recordings and extracts, representing the sound events from up to three sources of interest. The source categories, signal-to-noise ratios, and inter-onset characteristics of the sound events are drawn semi-randomly from the normal distributions. The corresponding parameters, as well as the overall sound level of each scene, are conditioned on a desired type of sound environment: quiet street, noisy street, very noisy street, park, or square. All background and event extracts appearing in the synthetic training set are recorded in the city of Lorient. The ground truth composition, i.e., separate channels for each active sound source, is known for the synthetic scenes. This enables automatically labeling the source presence to train the deep neural architecture in a supervised approach. the synthetic scene generation and automatic annotation processes are further detailed in Gontier *et al.* (2019). Figure 2 shows an example of the source identification by the trained model. Only the third-octave spectrogram of the mixed scene (Fig. 2, top) is visible to the network.

### D. Matching measurements to questionnaires

#### 1. Temporal match

Table IV represents the timeline of the different periods concerned by this study.

• The first questionnaire was sent out in early January 2019, and the responses poured in until early March 2019 (although most arrived within the first few weeks). This period, hereafter, will be called Q1;
• the second questionnaire was sent out in early April 2020, and the responses came in until mid-May 2020 (although most arrived within the first few weeks). This period, hereafter, will be called Q2; and
• because the measurements from the sensors are only available since the end of 2019, we chose the period between January 11 and February 11, 2020 as the “out of lockdown” period, called M1, and the period from April 11 to May 11, 2020 as the “during lockdown” period, called M2.

**TABLE IV.** The timeline of the time periods considered in the study to gather data.

	January 2019	March 2019	January 2020	February 2020	March 2020	Mid-April 2020	Mid-May 2020
French lockdown	Before lockdown				After lockdown		
Questionnaire	Q1					Q2	
Measurement			M1			M2	
Cross analysis	P1					P2	

The analyses are performed on the period out of lockdown, called P1, which will relate the results from the beginning of the year 2019 (Q1) to the beginning of the year 2020 (M1), assuming that the sound environments remain similar over these two time periods.

The period during the lockdown, called P2, relates the responses to the questionnaires (Q2) to the measurements (M2) over the same time period (April–May 2020).

#### 2. Spatial match

There are no direct spatial links that can be established between the sensors and questionnaires as the sensors were not positioned in the gardens or right in front of each house of the respondents. We, thus, propose to interpolate the results of the questionnaires and calculate an aggregated value for each of the sensor locations. For this purpose, a spatial Kriging algorithm is used, following the protocol used in Aumond *et al.* (2018).

The Kriging method is a well-known interpolation method, which has been used in a variety of applications, particularly in the environmental field. It bears resemblance with the classical data assimilation methods, which have been applied to environmental forecasting, particularly at the urban scale for air pollution and noise pollution. The approach is relevant when a meaningful function can fit the empirical variogram of a value to interpolate. The variogram and kriging algorithms presented in this study are applied using the “variogram,” “vgm,” and “gstat” functions of the gstat *R* package (Pebesma, 2004).

**FIG. 3.**
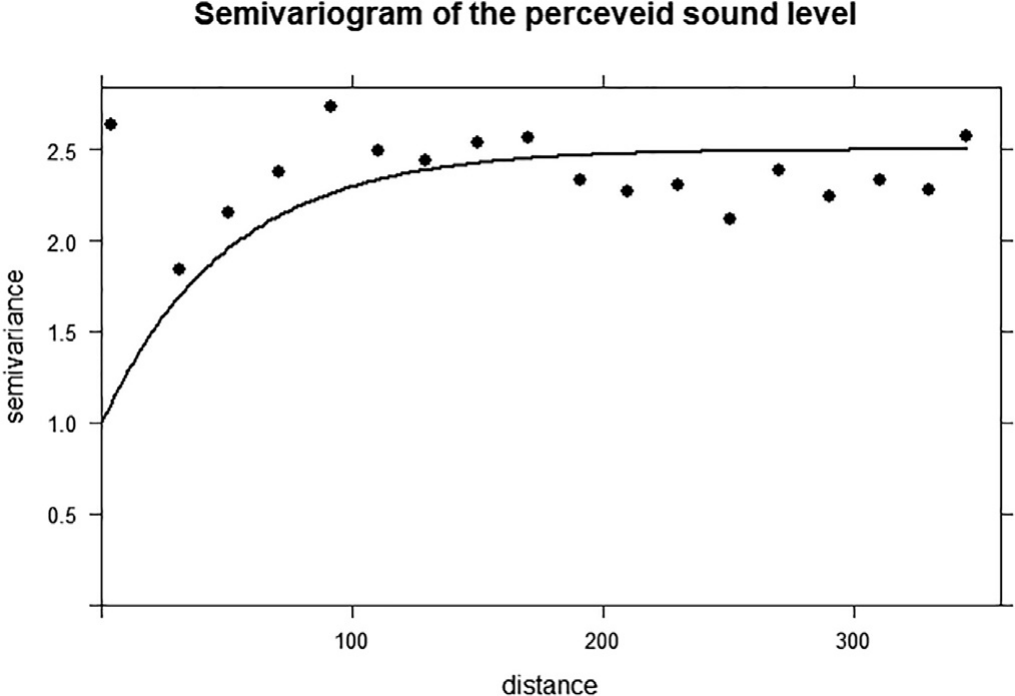
The empirical variogram of the Sil parameter along the Euclidian distance. The fitted parameters are nugget (1.0), range (50 m), and sill (1.5).

The parameter used to calculate the empirical variogram is the perceived sound level “Sil,” evaluated in the first part of the questionnaire. The variogram is calculated over a distance of 350 m. The exponential covariance model is used to calculate the best fit of the experimental variogram. This regression curve fitting estimates the three following standard parameters: “nugget” (1.0), “range” (50 m), and “sill” (1.5). Figure 3 present the empirical variogram and the fitted curve.

## III. RESULTS

### A. Perceptual analysis

It can be expected that the lockdown had a drastic impact on the overall sound levels and, also, on the sound sources activity in the urban sound environment, and we expect that this impact can be measured from the gathered data. Table V shows the variation on their respective scales of a set of questionnaire variables between January 2019 and March 2020 (respectively, Q1 and Q2). The statistical information is extracted from the two-sample nonparametric studentized permutation test for paired data (Brunner-Munzel test) from the *R* package “nparcomp” (Konietschke *et al.*, 2015). The Brunner-Munzel test (also called the generalized Wilcoxon test) is a nonparametric statistical test for the stochastic equality of two samples. The null hypothesis for the test is *H*0 = 0.5, which implies stochastic equality. If the estimated relative effect “hat” is superior to 0.5, greater values occur in the second group and conversely.

**TABLE V.** The Brunner-Munzel test (*n* = 31-Q1/31-Q2) between Q1 and Q2; hat, estimated relative effect; lower, lower limit of the confidence interval; upper, upper limit of the confidence interval; *T*, studentized test statistic; *p* val, *p*-value for the hypothesis. ***p-value < 0.01, **p-value < 0.05, *p-value < 0.1.

	Lower	Hat	Upper	*T*	*p* val		Lower	Hat	Upper	*T*	*p* val
Brd	0.71	0.80	0.89	6.89	^***^	Pl	0.81	0.89	0.97	9.93	^***^
Tra	0.06	0.17	0.28	−6.11	^***^	Ex	0.49	0.64	0.80	1.87	^**^
CaV	0.32	0.44	0.57	−0.91		Ca	0.75	0.85	0.95	6.98	^***^
ExV	0.07	0.18	0.29	−6.03	^***^	Ev	0.07	0.17	0.27	−6.83	^***^
ChV	0.26	0.41	0.57	−1.15		Sil	0.73	0.83	0.94	6.38	^***^
Gul	0.43	0.57	0.71	1.01		Ad	0.60	0.71	0.82	3.66	^***^
2Wh	0.12	0.27	0.43	−3.01	^***^						

As expected, we observe an important and significant decrease in the perceived sound level (Sil) as well as in the presence time of road traffic (Tra) and two-wheelers (2Wh). We also notice a significant increase in the time of presence of birds (Brd). Nevertheless, this result must be put into perspective as this increase can be due to the lockdown effects and also to the spring seasonal effect (P1 is in winter, and P2 is in spring). There is a significant decrease in the perceived time of presence of expressive voices (ExV); nevertheless the assessment of calm voices (CaV) did not change significantly.

More generally, the perceptual assessments allow a rather detailed description of what happened in the sound environment of Lorient as it mixes the affects and descriptions of the sound source activities. However, it is difficult to capture the subjective part of the phenomenon. For example, people probably spent more time passively or actively listening to the sound environment than in the first questionnaire and at different periods of the day. The variations in the evaluation may then come from changes in the mode of perception rather than from the sound environment *per se*. In addition, the low temporal resolution of the perceptual data makes it difficult to evaluate the impact of the moment at which the respondent does the evaluation and his/her ability to mentally project himself/herself on an average value of the period under evaluation.

The literature often mentions the perceptual models, which link two main perceptual dimensions (pleasantness and eventfulness) to the sound sources birds, traffic, and voices (perceived time of presence; Aumond *et al.*, 2017; Ricciardi *et al.*, 2015). A statistical analysis using the *R* package “multcomp” (Bretz *et al.*, 2010) allows us to extract the multilevel components of this type of model. It is a question of evaluating which part of the variance proportion in the model can be attributed to the individuals (associated with the different locations) and which part can be attributed to the analysis period (Q1/Q2). Table VI shows the results of the multilevel linear regressions for the independent variables pleasantness (Pl) and eventfulness (Ev).

**TABLE VI.** The multilevel linear regression of the independent parameters pleasantness (Pl) and eventfulness (Ev) and the dependent parameters presence time of birds (Brd), traffic (Tra), and calm voices (CaV) and expressive voices (ExV). The random effects are associated with the individuals or the questionnaire period (Q1/Q2). ***p-value < 0.01, **p-value < 0.05, *p-value < 0.1.

*n* = 62	Pl	Ev
Intercept	6.6***	2.0**
Brd	0.3***	0.0
Tra	−0.5***	0.3***
CaV	0.0	0.09
ExV	−0.1	0.11
	*R*² total = 67%	*R*² total = 37%
	*R*² fixed effects = 52%	*R*² fixed effects = 15%
Random effect :	Standard deviation	Standard deviation
Individuals	0.6	0
Questionnaire period (Q1/Q2)	0.1	0.7

The times of presence of birds and traffic have a significant impact over the estimation of the pleasantness. The random effect is quite small and mainly caused by the disparity between the individuals. The model and its strength are very close to the literature (Aumond *et al.*, 2017; Aumond and Lavandier, 2019; Ricciardi *et al.*, 2015). For example, for the city of Paris with quite different experimental conditions but with a similar questionnaire (Aumond *et al.*, 2017), the best perceptual model is

$$\text{Pl}=8.11-0.38^{*}\left(\text{OL}\right)-0.15^{*}\text{Tra}+0.20^{*}\text{Voi}+0.15^{*}\text{Brd},$$

where “Voi” is the perceived time of presence of the voices, and the overall loudness, “OL,” corresponds to (10 - Si) of this paper and explains 58% of the global variance (*R*_adj_^2^ = 58%). In Lavandier *et al.* (2021), the readers also can find more comparisons between these similar models on different study cases. The multilevel linear regression also reveals that the variation of the pleasantness depends much more on the individual differences (e.g., different locations) than on the differences in the different periods.

We find that the time of presence of traffic is the only one that has a significant impact on the estimation of the eventful character. The total explained variance is much lower (*R*^2^ = 37%). We can also observe a significant random effect due to the period of the questionnaire. This is in line with the fact that the Q2 period is much calmer than the Q1 period, and the calm dimension is opposed to the eventful dimension in the circumflex model (Axelsson *et al.*, 2012). The literature consistently highlights the instability of the definition of this parameter, according to the translations (Jeon *et al.*, 2018; Nagahata, 2018). For example, Jeon *et al.* (2018) state in their study that “The perceived dominance of sound of human activities shows a positive relationship with eventfulness scores in Korea and Sweden, while the same relationship is not statistically significant in France.” Also, because of the ambivalence of this term in French, a semantic shift may have occurred when there was very little traffic during the lockdown period.

### B. Sound level analysis

**FIG. 4.**
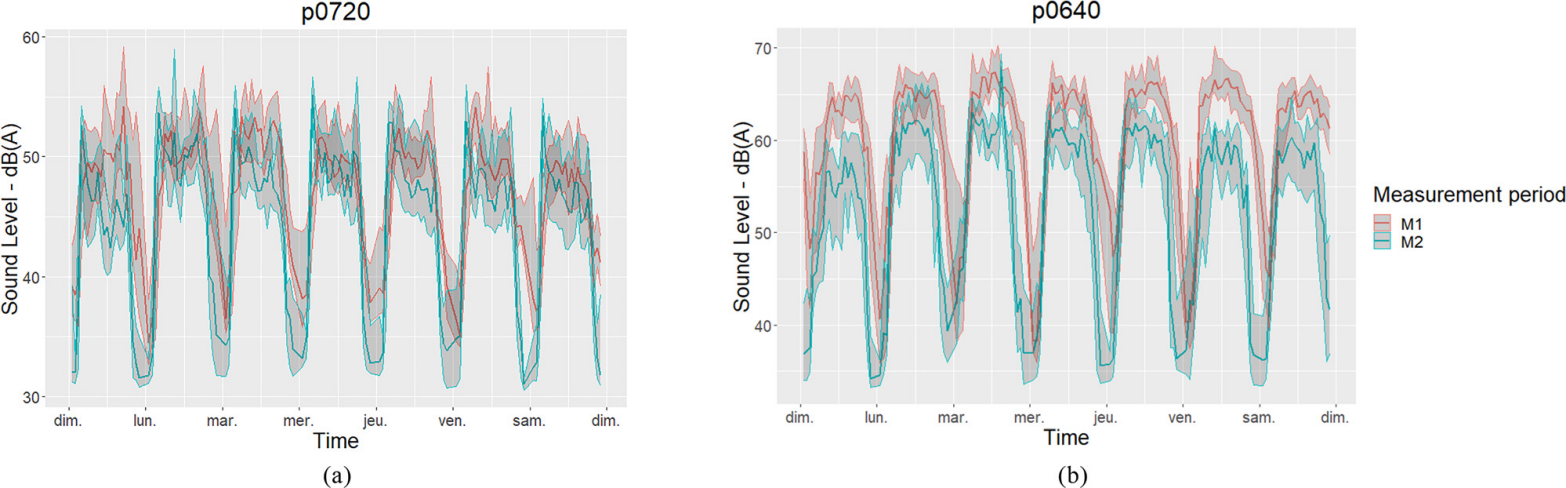
(Color online) The median and interquartile range of L_Aeq,1s_ (10 min, every hour) for the seven days of the week and two focus stations, respectively, located (a) in a quiet residential area and (b) on a boulevard for each period of measurement M1 and M2 are shown.

Figure 4 shows the median and interquartile range of L_Aeq,1s_ (10 min every hour corresponds to 600 values of L_Aeq, 1s_) for the seven days of the week and the two stations, respectively, located on a boulevard and in a quiet residential area for each period of measurement M1 and M2 (*n*_1h_ = 2400L_Aeq,1s_ values for 4 weeks).

**FIG. 5.**
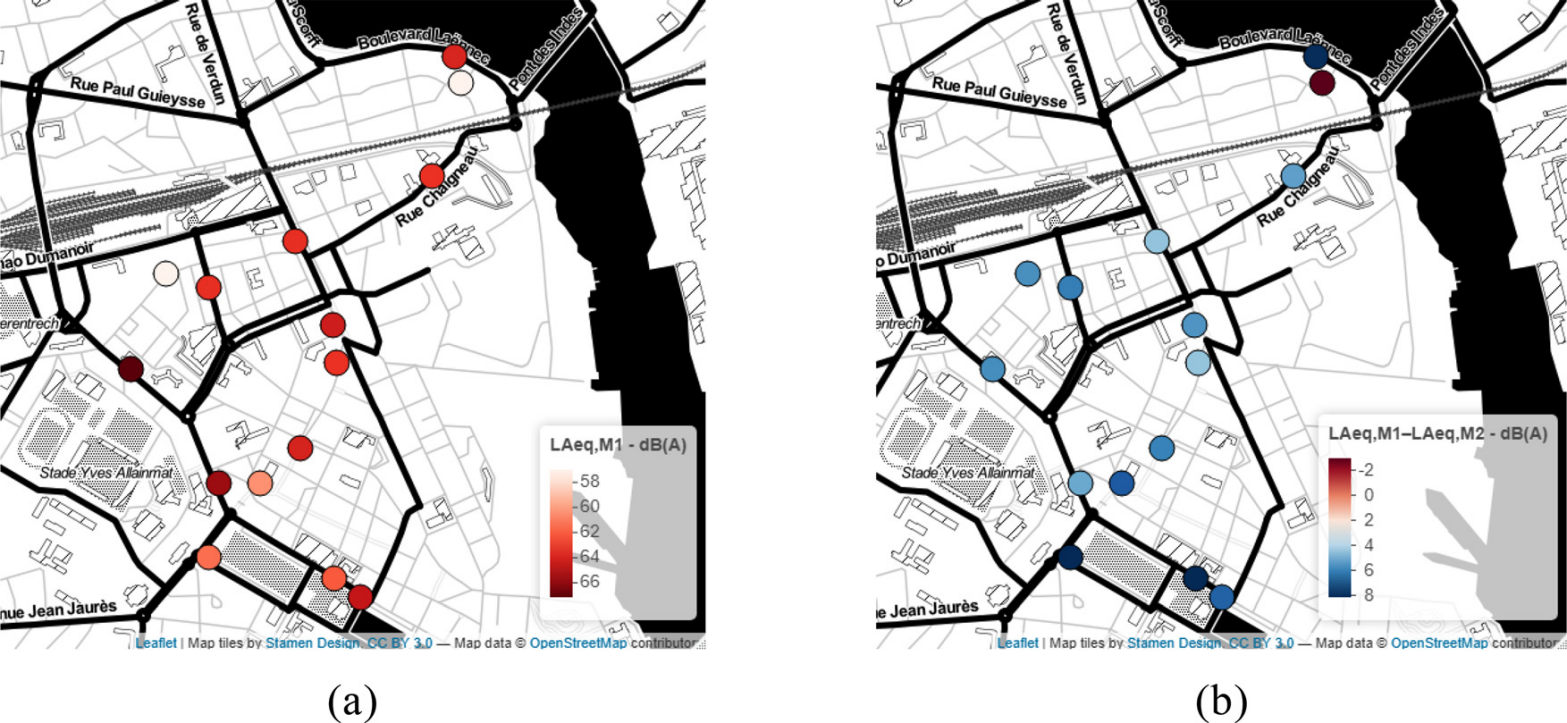
(Color online) For the 15 stations, (a) the pre-lockdown L_Aeq,M1_ and (b) its difference with the period during the lockdown L_Aeq,M1_ – L_Aeq,M2_ are depicted.

The typical daily and weekly patterns are maintained for both periods studied. A difference of up to −15 dB(A) is observed between the period before and during the lockdown for the station p0640. A multilevel linear regression (*n* = 470 992 groups, 15 stations) indicates that the fixed effect related to the period (M1 vs M2) is 7.4 dB(A) (standard deviation, 0.03; *t*-value, −279.70). This dramatic decrease is in accordance with the literature (Asensio *et al.*, 2020b; Bruitparif, 2020; Munoz *et al.*, 2020). The random effects related to the stations have a significant variance of 12 dB(A), which reflects a fairly large variability on the selected stations. Figure 5 shows the L_Aeq,M1_ computed from L_Aeq,1s_ (10 min every hour) and its difference (pre, L_Aeq,M1_ and during lockdown, L_Aeq,M1_ – L_Aeq,M2_) for the 15 stations.

The Pearson correlation coefficient, calculated between L_Aeq,M1_ and L_Aeq,M1_ – L_Aeq,M2_, shows that the most noise-exposed sensors are generally those with the greatest decrease in the noise level (*r* = 0.4, *p* < 0.01). The most drastic decrease was in the downtown area around Jules Ferry Park. It is the center of the city with a lot of animation, road traffic, bars, restaurants, etc. during the non-lockdown period. Only one sensor has seen its level slightly increase, and it is the sensor p0720 near the Scorff River, located in a residential area, possibly caused by an increase in the naturally occurring sounds and the presence of local residents walking during the M2 period in a very quiet area.

### C. Sound recognition analysis

**FIG. 6.**
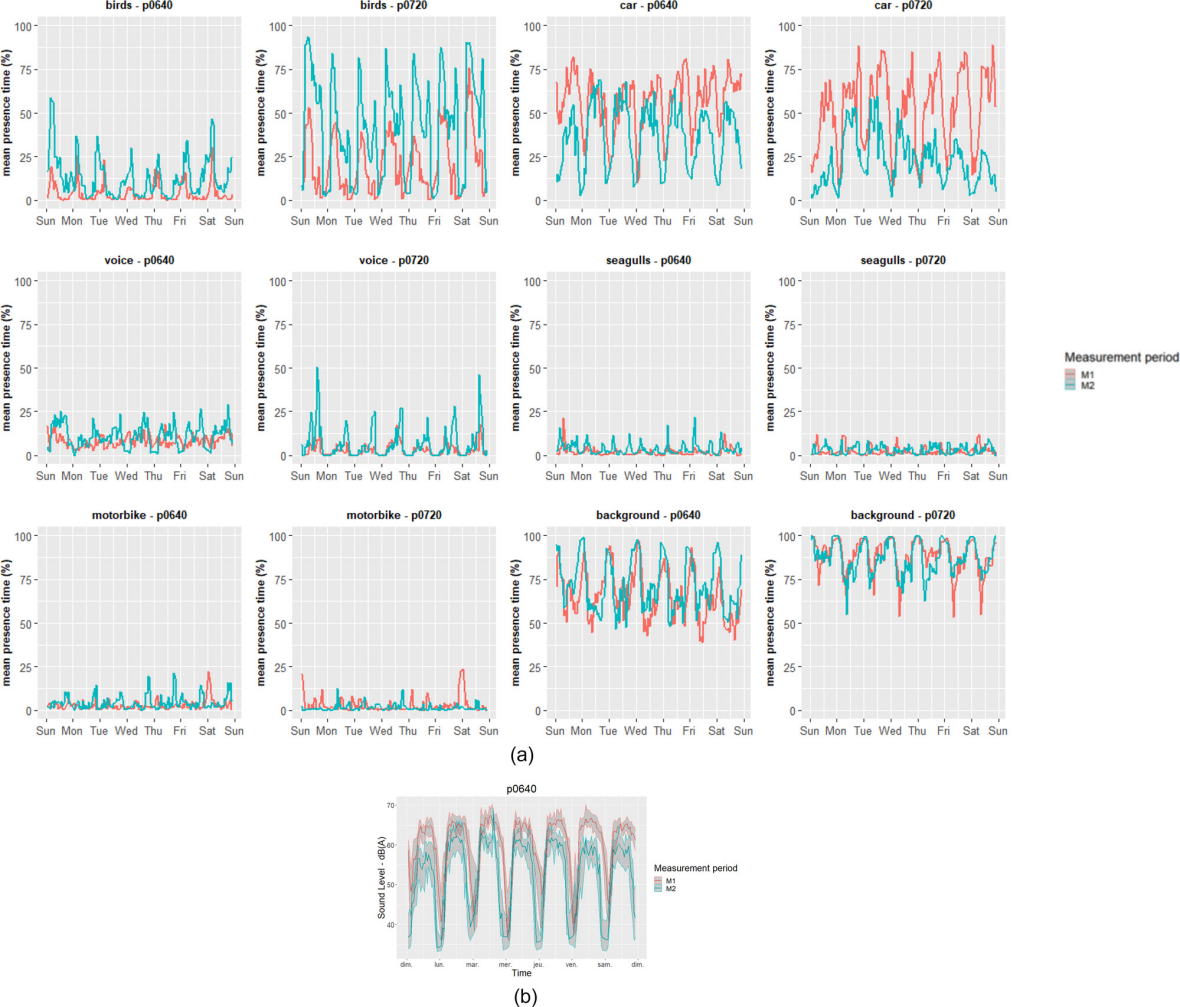
(Color online) The average hourly median of the presence time of the six estimated parameters for the two focus stations over the week period (left column, boulevard; right column quiet residential area) and the presence time of the six estimated parameters are shown.

Figure 6 shows the average hourly median of the perceived time of presence estimated for the six different parameters from the algorithm presented in Sec. II C for the seven days of the week and two stations. The two focus stations selected are again p0640 as a representative of a boulevard and p0720 as a representative of a residential area.

First, the expected general diurnal and weekly behaviors are observed for most of the variables (e.g., the morning bird songs peak at sunrise), which strengthen our confidence in the source recognition model. Concerning the voices, there is a significant increase during the afternoons of the M2 period and especially for the station in the quiet area and during the weekends. This is probably caused by the outings in the urban outdoor space (the lockdown rule in France allowed an exit of 1 h possible). In France, the weather was particularly good during this period, probably reinforcing this effect. The difference between the M1 and M2 periods is less noticeable on Wednesdays (in France, children have the afternoon off on this day) and Sundays. Concerning the motorcycles, in the M1 period, there was a peak on Friday and Saturday evenings. In the M2 period, this peak seems to occur more frequently at the end of the day. Several hypotheses, discussed in Sec. IV, can account for this phenomenon. As expected, the “background” parameter is inversely correlated with the number of events recognizable by the algorithm, and its level is generally higher in the M2 period and for the station located in the quiet area. Finally, birds are increasingly noticed during M2 at both locations, which is in accordance with Aletta *et al.* (2020, and Gordo *et al.* (2021).

**FIG. 7.**
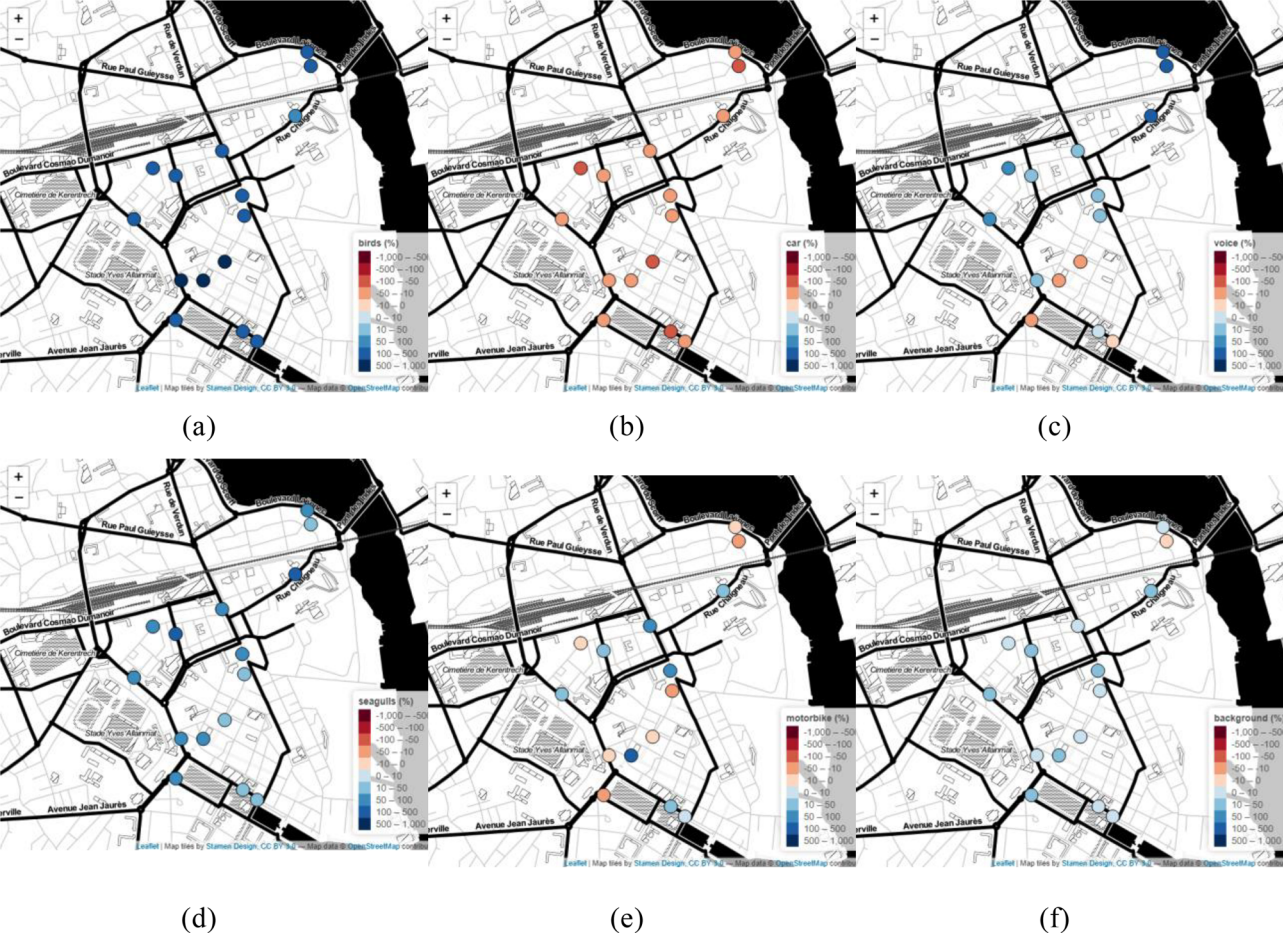
(Color online) The relative change between the average estimated time of presence of sound sources between the periods M1 and M2 (M2 - M1)/M1 is depicted.

We complete this analysis with a nonparametric *post hoc* test (Brunner-Munzel). Table VII shows the difference (and related significance) of the 6 estimated variables between M1 and M2. Also, Fig. 7 shows the relative change of the mean value during the periods M1 and M2 (reference period M1) for relevant estimated parameter and each station. Figures 6 and 7 and the statistical evaluation show a strong increase in the estimated presence time of birds and seagulls and a decrease in the presence time of road vehicles, recognized by the algorithm. There is also a decrease in the estimated presence time of voices during the lockdown period (M2) in the hyper center and near the bars but an increase in the small streets or on the ballade of the Scorff River. Figure 7(f) shows the slight increase in the background estimation during the M2 period everywhere except close to the Scorff River. Figure 7(e) shows spatial variability in the changes in the “motorbike” estimates, which is difficult to relate in an obvious way to any spatial features.

**TABLE VII.** The Brunner-Munzel test (*n* = 154/154); hat, estimated relative effect; lower, lower limit of the confidence interval; upper, upper limit of the confidence interval; *T*, studentized test statistic; *p* val, *p*-value for the hypothesis. ***p-value < 0.01, **p-value < 0.05, *p-value < 0.1.

	Lower	Hat	Upper	*T*	*p* val		Lower	Hat	Upper	*T*	*p* val
Bird	0.88	0.90	0.93	28.06	***	Seagulls	0.67	0.72	0.77	8.88	***
Car	0.11	0.14	0.18	−18.66	***	Motorbike	0.43	0.49	0.55	−0.35	
Voice	0.49	0.55	0.60	1.70	*	Background	0.56	0.60	0.64	5.28	***

### D. Cross-comparison of the different evaluation methods

#### 1. Preamble

All of the methods presented provide complementary information for the analysis of the change in the sound environment. In this section, we propose to cross-reference these data to highlight, or not, the consistency between these perspectives. Section II D presents the spatial and temporal methods used to link the measurement stations to the different questionnaires.

#### 2. Global analysis

First, the comparison of the results of Tables V and VII shows a very good agreement between the decrease in the source presence as measured by the questionnaires and automatic source recognition tools for birds (hat values of 0.8 and 0.9) and road traffic (hat values of 0.14 and 0.17).

The analysis for voice is less straightforward. The questionnaire introduced a discrimination between the expressive voices, calm voices, and children voices, which is not accessible through the sound recognition model. The algorithm learning database is mainly conversational voices, and one can assume that the algorithm refers primarily to calm voices. Then, both forms of analysis converge on a very small change in the calm voices times of presence.

Last, the decrease in the presence of motorcycles is much higher for the questionnaires than in the source recognition estimates. A first hypothesis would be that when the traffic noise is low, the noise of the 2Wh propagates further before being masked. Thus, the estimated presence time of this parameter decreases only slightly from the algorithm point of view, but the perceived time of presence by the inhabitants decreases more strongly due to the decrease in the number of vehicles.

Another hypothesis could be that the type of two-wheeled noise sources and driver behavior may have changed between the two periods. Many delivery vehicles appeared during the P2 period (small engines and moderate acceleration), and there may have been a decrease in the recreational or long-distance use of 2Wh. The recognition algorithm does not identify the difference between these two types of vehicles, but their noise characteristics and the perception induced for the inhabitants may be very different.

#### 3. Spatial analysis

Using the method described in Sec. II D, it is possible to interpolate the perceived values at the locations of each of the sensors in the area. Then, the spatiotemporal correlations between the sensor estimates and questionnaires for the P1 and P2 periods and each location can be performed. Table VIII shows the Pearson correlation coefficients with their respective significances (the *p*-value is inferior to certain thresholds) among the variables from the questionnaire, the source recognition, and also the physical indicators directly calculated from the sensors.

**TABLE VIII.** The Pearson correlation coefficients between the perceived (interpolation), physical, and source recognition parameters for the periods P1, P2, and P1 and P2. ***p-value < 0.01, **p-value < 0.05, *p-value < 0.1. Bold entries are correlation coefficients with a *p*-value < 0.1.

Perceptual assessments	Recognized times of presence	Physical indicators	P1 (spatial) *n* = 15	P2 (spatial) *n* = 15	P1 and P2 (spatiotemporal) *n* = 30
Sil		L_Aeq_	−0.36	−0.44*	−0.78***
Sil		L_A50_	−0.37	−0.51**	−0.83***
Tra		L_Aeq_	0.34	0.35	0.76***
Tra		L_A50_	0.32	0.18	0.81***
	Car	L_Aeq_	0.34	0.65***	0.63***
	Car	L_A50_	0.26	0.44*	0.56***
Brd	Birds		0.30	−0.23	0.63***
Tra	Car		−0.08	0.34	0.47***
CaV	Voice		0.53***	−0.01	0.27
ExV	Voice		0.72***	0.13	0.19
ChV	Voice		0.51**	−0.44*	0.20
Gul	Seagulls		0.33	−0.20	0.52***
2Wh	Motorbike		0.28	0.04	−0.01
Sil	Background		0.46**	0.09	0.37***

First, it should be pointed out that these correlation coefficients reflect a link between the observed variables and also the spatial and/or temporal dynamics of these variables. The weak dynamics will probably lead to a more difficult link to highlight between two variables.

The indicators L_Aeq_ and L_A50_ correspond to the average values of all of the L_A50,10min_, L_Aeq,10min_ calculated, respectively, on the periods P1 and P2. As expected, the perceived silence (Sil) and measured sound level indicators L_Aeq_ and L_A50_ are significantly inversely correlated (*r*_Sil/LAeq_ = −0.78; *r*_Sil/LA50_ = −0.83). These variables for the periods P1 are significantly inversely correlated (*r*_Sil/LA50_ = –0.36; *r*_Sil/LA50_ = −0.37) and P2(*r*_Sil/LA50_ = −0.44; *r*_Sil/LA50_ = −0.51). The equivalent or median sound level is again a good physical indicator of the perceptual variable “silent to noisy.” The correlation coefficients may seem rather weak, but the perception of the residents is based on the memory and not on a judgment of a sound environment that they listen to. Also, the low density of the sensors analysed in the area and, therefore, to the large distances between the sensors and questionnaires can explain these low values.

Exactly the same dynamics are observed when comparing the measured sound levels L_Aeq_ and L_A50_ and the perceived traffic presence time (Tra). We also see that the correlations are slightly weaker for this perceived variable, which reflects the part of the overall noise level that is not related to road traffic. The gap between the time of presence of road traffic and the noise level is confirmed by looking at the correlation coefficients between the noise levels and the presence time of cars as estimated by the algorithm at each sensor (*r*_cars/LA50_ = 0.63; *r*_cars/LA50_ = 0.56).

Even if the correlation coefficients between the perceived times of presence of traffic and birds (recognized by the algorithm and perceptually assessed) is not significantly spatially correlated over the periods P1 (*r*_Brd/Birds_ = 0,30; *r*_Tra/cars_ = −0,08) and P2 (*r*_Brd/Birds_ = −0.23; *r*_Tra/cars_ = 0.34), the spatiotemporal correlation coefficients are significant for these two observables (*r*_Brd/Birds_ = 0.63; *r*_Tra/cars_ = 0.47), which confirms that the perceptual and algorithmic methods lead to the same observations.

The perceived time of presence of voices is significantly correlated to the estimation from the source recognition only for the periods P1 (*r*_CaV/Voice_ = 0.53; *r*_ExV/Voice_ = 0.72; *r*_ChV/Voice_ = 0.51). During the period P2, the people were restricted to go out in a radius of 1 km around their home. The resulting loss of spatial dynamics for this variable, thus, may prevent the measurement/perception correlation from being revealed.

Last, we observe a significant spatial correlation on the period P1 of the perceived sound level Sil and the variable background of the algorithm (*r*_Sil/Background_ = 0.46). On the P2 period, the dynamics of this last variable are probably too weak to reveal any difference (*r*_Sil/Background_ = 0.09). Over the period P1 + P2, the spatiotemporal correlation of this variable is significant (r_Sil/Background_ = 0.37). This variable, therefore, seems to correctly reveal the absence of the sound sources in the environment.

## IV. DISCUSSION

First of all, the overall results are consistent with the existing literature: we observe an overall decrease in the noise levels in comparable ranges with respect to those of other cities, and this decrease is all the more important near the main roads. We also observe an emergence of natural sound sources and a drastic decrease in mechanical noise, which are both reflected by the questionnaires and sensors.

When crossing the physical and perceptual analysis methods, two main limitations arise. The small number of sensors spatially close to the participant questionnaires does not allow a direct link between a questionnaire and a nearby measurement station. The chosen interpolation method alleviates this issue but introduces approximations. Nevertheless, the average proximity between the stations and questionnaires remains acceptable to justify the methodology (median = 108 m, 80% < 155 m). From a temporal point of view, the technical limitations forced us to choose the periods spaced one year apart for the unconfined period (M1 vs Q1). Despite these approximations, the proposed protocol allows us to evaluate the performance of the source recognition algorithm on site and at scale with an applied scenario and confront its prediction with the citizens' appreciation.

Regarding the questionnaire and cofactors influencing the perception, an important aspect of the housing questions is whether the respondents live alone or with others in their homes, as investigated in Torresin *et al.* (2022). This question is particularly relevant in the context of the COVID-19-related lockdown. Unfortunately, this variable was not collected in our questionnaire.

Our analysis of the perceptual results indicates that the individuals/places are more important in the evaluation of pleasantness than the difference between the periods. In a context such as the lockdown with very high variation in the sound environment, even in the most unpleasant locations in the city, one could hypothesize that this observation also partly reflects the non-acoustic factors (e.g., visual context; Jeon and Jo, 2020; Jeon *et al.*, 2011) that cannot be captured by the sensors (and, thus, by the model).

In this study, the perceptual assessment provides a fairly accurate description of what happened in the Lorient sound environment during the lockdown period. We could hypothesize that there is no bias in the perceptual assessment and it accurately reflects the sound environment. But the lockdown period could have implied a modification of the perceptions, such as an overestimation of the time of presence of the birds, the decrease in the road noise exacerbated by the sensations of the moment, or the surprise to discover such a peaceful urban sound environment. It would, therefore, be interesting to go further by conducting a contextual analysis or an analysis according to the psychosocial characteristics of the individuals interviewed, but the sample size of our study was not sufficient.

Different from that of the questionnaire, which can induce biases in the comparison, the method linked to the source recognition allows a very detailed temporal follow-up. For example, the low correlation coefficient between the perceived time of presence of the 2Wh and the measurements can be partly explained by the very different temporal resolutions of these two observation methods. Indeed, the presence of the 2Wh has a very specific behavior, in particular at the time of the lockdown in the evening between 6 pm and 8 pm, which can probably be attributed to food deliveries. It is difficult to estimate how this behavior translates into perceptual assessments over the period linked to the questionnaire.

## V. CONCLUSION

In this paper, three methods of the sound environment analysis were jointly considered over the periods before and during the lockdown related to the COVID-19 crisis in the city of Lorient, France. The analysis of the sound levels reflects the results of many other studies on the subject, namely, an average decrease in the equivalent sound level of about 5–10 dB(A). The analysis of the questionnaire results allows us to go into detail about the changes in the perceived times of presence of certain sources, such as those expected, for instance, the drastic decrease in the road traffic and th increase in the birds' songs perceived presence.

This study also introduced a protocol of integration of the analysis of the presence of sound sources from questionnaires and automatic estimation using the deep convolutional network algorithms. The analysis of the latter reveals a temporal detail on the presence of sources, which is very complementary to the questionnaires. The differences between the questionnaires and the algorithm also make it possible to question the perceptual or algorithmic biases that may be present.

The lockdown related to COVID-19 allowed us to validate the relevance of the proposed approach as strong assumptions on the expected behaviors of the analysis variables could be made. Despite some methodological and technical limitations that could be improved in the future, this paper demonstrates the interest of introducing multidisciplinary analyses, as proposed in the triangulation section of the ISO/TS (2019) soundscape standard, to account for the short-term and long-term evolutions of the urban sound environments and their appraisals. With further validation to increase our confidence in the performance of the recognition algorithm, we believe that this kind of cross-analysis will allow us to highlight the perceptual biases, which are particularly revealing of the modes of perception of the sound environments considered by the citizens.

## Footnotes

^1^
The full code and databases will be made available upon publication.

^2^
See https://bitbucket.org/mlagrange/simscene (Last viewed 1/24/2022).
